# Iatrogenic Ulnar Nerve Neuropathy Following Reverse Total Shoulder Arthroplasty: Electrodiagnostic Findings in 18 Patients

**DOI:** 10.7759/cureus.39089

**Published:** 2023-05-16

**Authors:** Lisa B Shields, Vasudeva G Iyer, Yi Ping Zhang, Christopher B Shields

**Affiliations:** 1 Neurological Surgery, Norton Neuroscience Institute, Norton Healthcare, Louisville, USA; 2 Clinical Neurophysiology, Neurodiagnostic Center of Louisville, Louisville, USA

**Keywords:** ultrasound study of nerve, electrodiagnostic studies, iatrogenic nerve injury, reverse total shoulder arthroplasty, ulnar nerve neuropathy, orthopedics, neurology

## Abstract

Background

A reverse total shoulder arthroplasty (RTSA) is often recommended for rotator cuff pathology and may be associated with a myriad of complications, including prosthetic instability, infection, humeral problems, and glenoid loosening. Neurological injuries following an RTSA are infrequent and are usually related to brachial plexus or proximal nerve injury in the affected arm. Iatrogenic ulnar nerve neuropathy is exceedingly rare.

Aims

This study describes the clinical and electrodiagnostic (EDX) features of 18 patients with ulnar nerve neuropathy complicating RTSA.

Materials and methods

All patients underwent EDX studies, and 14 had an ultrasound (US) study.

Results

All patients complained of numbness, tingling, hyperalgesia, and/or allodynia in the distribution of the ulnar nerve. Eight (44%) patients reported hand weakness, and one (6%) noted wasting of the intrinsic hand muscles. Decreased pinprick sensation in the ulnar nerve distribution was detected in all patients. Seventeen (94%) patients had weakness of the ulnar nerve-innervated intrinsic hand muscles. All patients had focal slowing of the motor conduction of the ulnar nerve across the elbow. Sensory potentials were either absent or of a low amplitude over the digital and/or dorsal cutaneous branch of the ulnar nerve in all patients. Twelve (86%) patients showed an increase in the cross-sectional area (CSA) of the ulnar nerve at the elbow; six (43%) had a hypoechoic ulnar nerve. Ulnar nerve neuropathy was confirmed at the elbow in all 18 patients. Of the 14 (78%) patients who underwent surgical intervention for ulnar nerve neuropathy following an RTSA, only four had complete symptom resolution.

Conclusions

Surgeons should be cognizant of ulnar nerve neuropathy as a potential complication of an RTSA and take precautions to avoid damage to the ulnar nerve intraoperatively. EDX and US studies should be performed to confirm and assess the site and severity of the injury.

## Introduction

Developed in the late 1980s and approved by the US Food and Drug Administration in April 2004, the reverse total shoulder arthroplasty (RTSA) reverses the shoulder joint by fixing a metal ball to the glenoid and presenting a spherical socket into the proximal end of the humerus [1,2]. The center of rotation is moved distally and medially to decrease the torque at the glenoid and uses deltoid muscle fibers instead of rotator cuff muscles to allow arm flexion [2-4]. This position creates both arm lengthening and the elongation of the brachial plexus [3]. Representing 64% of all shoulder arthroplasties, the RTSA is indicated for rotator cuff tear arthropathy, proximal humerus fractures, infection, tumor resection, and failed shoulder arthroplasty [1,2,5-7]. Intraoperative and early postoperative complications of this procedure include neurological injury, hematoma, periprosthetic infection, scapular notching, prosthetic fracture/instability/dislocation, mechanical baseplate failure, acromial/scapular fractures, glenoid component loosening/dislocation, and venous thromboembolism [1,2,5-8].

The incidence rates of nerve injuries during RTSA usually range from 1% to 4% clinically, although it may be as high as 70% when diagnosed electrodiagnostically instead of clinically [1,3-12]. Neurological injury may result from intraoperative traction due to surgical lengthening of the arm, intraoperative positioning, manipulation of the arm, compression secondary to retractor placement, and damage due to interscalene nerve blocks. RTSA-related neurological injuries are underreported; subclinical neurological injuries with postoperative electromyography (EMG) changes may be detected after this surgery; however, clinical evidence of significant neurological injury is rare [5]. During the exposure of the glenoid, the humerus is retracted posteriorly, externally rotated, and abducted, which may increase traction across the brachial plexus and peripheral nerves in anatomic proximity such as the axillary nerve [1,4,5,10]. While most neurological complications of RTSA are localized to the shoulder region (injury to the brachial plexus, musculocutaneous nerve, suprascapular nerve, and axillary nerve) [1,2,5,7,11], distal peripheral neuropathy (involving the median, ulnar, or radial nerve) may also occur [11]. The incidence of distal peripheral nerve injury from RTSA ranges from 0.9% to 5.2% [11]. Neurological injuries are more frequent in RTSA than total shoulder arthroplasty (TSA) due to arm lengthening and increased glenoid exposure in the former [4,9,10]. While most neurological injuries are transient neuropraxias, which resolve spontaneously, others may cause persistent pain and neurological dysfunction and require additional surgery such as an ulnar nerve transposition or cubital tunnel release (CBTR) [2,4,7,13]. Motor dysfunction lasting more than six weeks in duration associated with axonal degeneration detected by EMG studies indicates a more severe lesion and poor prognosis [13].

In this report, we describe the clinical symptoms of 18 patients with iatrogenic ulnar nerve neuropathy related to RTSA, all of whom underwent electrodiagnostic (EDX) studies and 14 of whom had ultrasound (US) studies. The presenting symptoms, clinical and EDX findings, and US features are presented. The mechanism of an ulnar nerve injury complicating RTSA is discussed.

## Materials and methods

Focused neurological examination

Under an Institutional Review Board (IRB)-approved protocol, we performed a six-year (from February 20, 2017, to April 18, 2023) retrospective analysis of patients referred to our neurodiagnostic center for EDX studies to evaluate the presence of sensory abnormalities and weakness in the distribution of the ulnar nerve. The patients underwent our protocol of a focused neurological examination of the upper extremities followed by nerve conduction velocity (NCV) and EMG studies. The clinical evaluation included documenting muscle strength, tone, and reflexes, as well as sensory examination to map out the topography and pattern of sensory loss. The presence of atrophy of the intrinsic hand muscles, clawing of fingers, Wartenberg sign, and similar features was documented.

Electrodiagnostic studies

The EDX studies were performed in our American Association of Neuromuscular & Electrodiagnostic Medicine (AANEM)-accredited facility using the standard protocol of our laboratory [14]. The ulnar nerve was studied by placing the recording electrode over the abductor digiti minimi (ADM) and the first dorsal interosseous (FDI) muscle. The digital and the dorsal cutaneous sensory branches were studied by the antidromic stimulation of the ulnar nerve at the distal forearm.

Ultrasound studies

A US study was conducted using the GE LOGIC e system (GE HealthCare, Chicago, IL) and a 12-18 MHz probe tracing the ulnar nerve. The cross-sectional area (CSA) of the nerve was measured along the course of the ulnar nerve at the wrist, proximal forearm, elbow, and distal upper arm.

Inclusion and exclusion criteria

The inclusion criteria included (1) onset after RTSA of sensory and/or motor symptoms (pain, paresthesia, numbness, or weakness) and documentation of objective sensory loss and/or muscle weakness in the distribution of the ulnar nerve and (2) EDX findings confirming ulnar nerve neuropathy (slowing of motor conduction of the ulnar nerve across the cubital tunnel and absent or decreased amplitude of sensory nerve action potentials {SNAP} of the ulnar nerve) and/or denervation changes during needle EMG. Patients with clinical or EDX findings suggestive of brachial plexopathy or polyneuropathy were excluded. Several metrics were collected including age, gender, symptom laterality (right/left), clinical history, interval between RTSA and EDX studies, findings on neurological examination, follow-up (ulnar nerve surgery or no treatment), EDX findings, and US features.

Institutional Review Board approval of research

The University of Louisville IRB determined that our study was exempt according to 45 CFR 46.101(b) under Category 4. The IRB number is 22.0880.

## Results

Clinical findings and neurological examination

A total of 18 patients were diagnosed with ulnar nerve neuropathy after an RTSA based on the presenting symptoms, findings on neurological examination, and EDX studies (Table 1).

**Table 1:** Demographics, Presenting Symptoms, Neurological Examination, and Follow-Up of Patients With Ulnar Nerve Neuropathy Following Reverse Total Shoulder Arthroplasty Symptoms: (A) numbness, tingling of digits five and four, or both; (B) weakness of the hand; (C) hyperalgesia/allodynia in ulnar nerve distribution; and (D) pain at the elbow Signs: (E) the loss of pinprick/light touch in ulnar nerve distribution and (F) the weakness of FDI, ADM, FDP(U), FCU R, right; L, left; M, male; F, female; f/u, follow-up; CBTR, cubital tunnel release; RTSA, reverse total shoulder arthroplasty; TSA, total shoulder arthroplasty; EDX, electrodiagnostic; FDP(U), flexor digitorum profundus (ulnar nerve-innervated part of FDP); ADM, abductor digiti minimi; FDI, first dorsal interosseus; FCU, flexor carpi ulnaris

Patient Number	Age (Years)/Gender	Side of Surgery (R/L)	Duration Between Shoulder Surgery and EDX	Presenting Symptoms at EDX	Neurological Examination at EDX	Treatment and Follow-Up
1	57/M	R	5 months	A and B	E and F	CBTR and the transposition of the ulnar nerve and anterior interosseous nerve to the motor branch of the ulnar nerve eight months after RTSA, f/u two weeks after CBTR: unchanged sensory function dorsum of the hand and two-point discrimination from before CBTR and the transposition of ulnar nerve
2	71/M	R	3 months	A and B	E and F	CBTR and the transposition of the ulnar nerve and anterior interosseous nerve to the motor branch of the ulnar nerve six months after RTSA, last f/u 20 months after CBTR: resolved symptoms except for poor little finger adduction
3	59/M	R	7 weeks	A	E and F	CBTR and the transposition of the ulnar nerve and anterior interosseous nerve to the motor branch of the ulnar nerve three months after RTSA, last f/u one month post-op: resolved symptoms
4	70/F	L	6 months	A, B, C, and D	E and F	Cushion to support the elbow after shoulder surgery, home exercises, and cortisone injection of R shoulder 17 months after RTSA, last f/u 17 months after reverse TSA: continued decreased sensation/burning of digits four and five
5	81/M	L	4 months	A	E and F	Ulnar nerve decompression six months after RTSA, last f/u nine months after ulnar nerve decompression: symptoms resolved
6	72/M	L	10 months	A	E and F	No treatment after RTSA, last f/u nine months after RTSA: continued numbness/tingling
7	64/M	R	3 months	A and B	E and F	Ulnar nerve decompression with submuscular transposition four months after RTSA, last f/u 24 months after ulnar nerve decompression: continued numbness/tingling of digits four and five
8	77/F	R	4 months	A, B, and D	E	Ulnar nerve decompression 20 months after RTSA, last f/u 23 months after ulnar nerve decompression: symptoms resolved
9	72/M	L	4 months	A and B	E and F	Ulnar nerve decompression and transposition and anterior interosseous nerve to the motor branch of the ulnar nerve seven months after RTSA, last f/u four months after ulnar nerve decompression: continued numbness of digits four and five
10	70/M	L	3 months	A	E and F	Ulnar transposition five months after RTSA, last f/u 13 months after ulnar transposition: symptoms resolved
11	68/M	L	3 months	A	E and F	CBTR eight months after RTSA, last f/u 11 months after CBTR: continued numbness/tingling
12	47/M	R	1 month	A and D	E and F	Ulnar nerve transposition 1½ months after RTSA; spinal cord stimulator placed for pain control 52 months after ulnar nerve transposition
13	66/M	R	5 weeks	A, B, C, and D	E and F	Ulnar nerve decompression 1½ months after RTSA, last f/u 16 months after ulnar nerve decompression: continued hypersensitivity to light touch with decreased motor function of ulnar nerve distribution
14	78 M	R	6 months	A and B	E and F	Home exercises, last f/u eight months after RTSA: continued numbness/tingling of digits four and five
15	73 M	L	12 months	A	E and F	CBTR and ulnar nerve transposition 18 months after RTSA, last f/u 14 months after CBTR/ulnar nerve transposition: continued numbness/tingling of digits four and five
16	69 M	L	10 months	A	E and F	No treatment after RTSA and continued numbness/tingling of digits four and five 14 months post-op, last f/u 17 months after reverse TSA: symptoms resolved
17	78 F	L	1 month	A	E and F	Ulnar nerve transposition two months after RTSA, last f/u six months after ulnar nerve transposition: improving numbness to 75% normal and continued weakness of intrinsic hand muscles
18	61 M	R	4 months	C and D	E and F	Ulnar nerve transposition six months after RTSA, last f/u nine months after ulnar nerve transposition: continued numbness and weakness of intrinsic hand muscles

The mean age was 68 years (range: 47-81 years), and the majority (15 {83%}) of patients were male. The occurrence of the ulnar nerve neuropathy was equally divided between the right and left sides, always on the side of the RTSA. The mean duration between the RTSA and the EDX studies was 4.5 months (5 weeks-12 months).

All patients experienced numbness, tingling, hyperalgesia, and/or allodynia in the distribution of the ulnar nerve on the side of the RTSA. Eight (44%) patients complained of hand weakness, and one (6%) noted muscle wasting of the hand. Five (28%) patients complained of elbow pain. On neurological examination, decreased pinprick sensation in the ulnar nerve distribution was noted in all patients. Seventeen (94%) patients had weakness of the ulnar nerve-innervated intrinsic hand muscles.

Surgical details

All patients underwent an RTSA. They were administered a shoulder scalene block prior to general endotracheal anesthesia. Seventeen (94%) patients were placed in the modified beach chair position during the RTSA, while one (7%) was performed in the lateral decubitus position.

Electrodiagnostic studies

The findings of the EDX studies are summarized in Table 2.

**Table 2:** Electrodiagnostic and Ultrasound Studies of Patients With Ulnar Nerve Neuropathy Following Reverse Total Shoulder Arthroplasty NCV of the ulnar nerve: (A) motor conduction velocity across the elbow of <49 m/s with normal conduction distally and proximally, (B1) CMAP amplitude over abductor digiti minimi (ADM)/first dorsal interosseous (FDI) of <2.5 mV, (B2) 50% or more drop in the amplitude of CMAP between stimulation at the wrist and at the elbow, (C) absent/low amplitude (<0 µV) SNAP digit 5/4, and (D) absent/low amplitude (<5 µV) SNAP dorsal cutaneous branch Needle EMG abductor digiti minimi (ADM)/first dorsal interosseous (FDI): (E) denervation (fibrillations/positive sharp waves), (F) decreased/absent motor unit recruitment, (G) large-amplitude, wide-duration polyphasic motor units, and (normal) normal recruitment L, left; R, right; CSA, cross-sectional area; SNAP, sensory nerve action potential; EMG, electromyography; NCV, nerve conduction velocity; SNAP, sensory nerve action potential; CMAP, compound motor action potential

Patient Number	Nerve Conduction Studies	Needle EMG	Ultrasound	Impression
1	A and C	F and G	Hypoechoic ulnar nerve at the elbow with increased CSA	R ulnar nerve neuropathy at the elbow with focal demyelination and axon loss
2	A, B1, B2, and C	E, F, and G	Hypoechoic ulnar nerve at the elbow with increased CSA	R ulnar nerve neuropathy at the elbow with focal demyelination, partial conduction block, and axonal loss
3	A, B1, B2, C, and D	E and F	Not done	R ulnar nerve neuropathy at the elbow with focal demyelination, partial conduction block, and axonal loss
4	A, B2, C, and D	F and G	Normal	L ulnar nerve neuropathy at the elbow with focal demyelination, conduction block, and axon loss
5	A and C	F and G	Not done	L ulnar nerve neuropathy at the elbow with focal demyelination and axon loss
6	A, C, and D	F and G	Hypoechoic ulnar nerve at the elbow with increased CSA	L ulnar nerve neuropathy at the elbow with focal demyelination and axon loss
7	A, B1, B2, C, and D	E, F, and G	Increased CSA of the ulnar nerve at the elbow	R ulnar nerve neuropathy with axonal involvement and conduction block at the elbow
8	A, C, and D	Normal	Not done	R ulnar nerve neuropathy at the elbow with focal demyelination and sensory axon loss
9	A, B1, C, and D	E, F, and G	Increased CSA of the ulnar nerve at the elbow	L ulnar nerve neuropathy at the elbow with focal demyelination and axon loss
10	A, B1, C, and D	E, F, and G	Increased CSA of the ulnar nerve at the elbow	L ulnar nerve neuropathy at the elbow, with focal demyelination and axon loss
11	A, B1, B2, C, and D	E, F, and G	Hypoechoic ulnar nerve at the elbow with increased CSA	L ulnar nerve neuropathy at the elbow with focal demyelination, partial conduction block, and axon loss
12	A, B1, B2, C, and D	E, F, and G	Not done	R ulnar nerve neuropathy at the elbow, focal and anterograde demyelination, conduction block, and axon loss
13	A, B1, B2, C, and D	E, F, and G	Increased CSA at the elbow	R ulnar nerve neuropathy at the elbow with focal demyelination, partial conduction block, and axon loss
14	A, B1, C, and D	E, F, and G	Hypoechoic ulnar nerve at the elbow with increased CSA	R ulnar neuropathy at the elbow with focal demyelination and axon loss
15	A, C, and D	F and G	Hypoechoic ulnar nerve at the elbow with increased CSA	L ulnar neuropathy at the elbow with focal demyelination and axon loss
16	A, C, and D	F and G	Hypoechoic ulnar nerve at the elbow with increased CSA	Left ulnar neuropathy at the elbow with focal demyelination and axon loss
17	A, B1, B2, C, and D	F	Hypoechoic ulnar nerve at the elbow with increased CSA	L ulnar neuropathy at the elbow with focal demyelination, conduction block, and sensory axon loss
18	A, B1, C, and D	E, F, and G	Increased CSA of the ulnar nerve at the elbow	L ulnar neuropathy at the elbow with focal demyelination and axon loss

**Figure 1:**
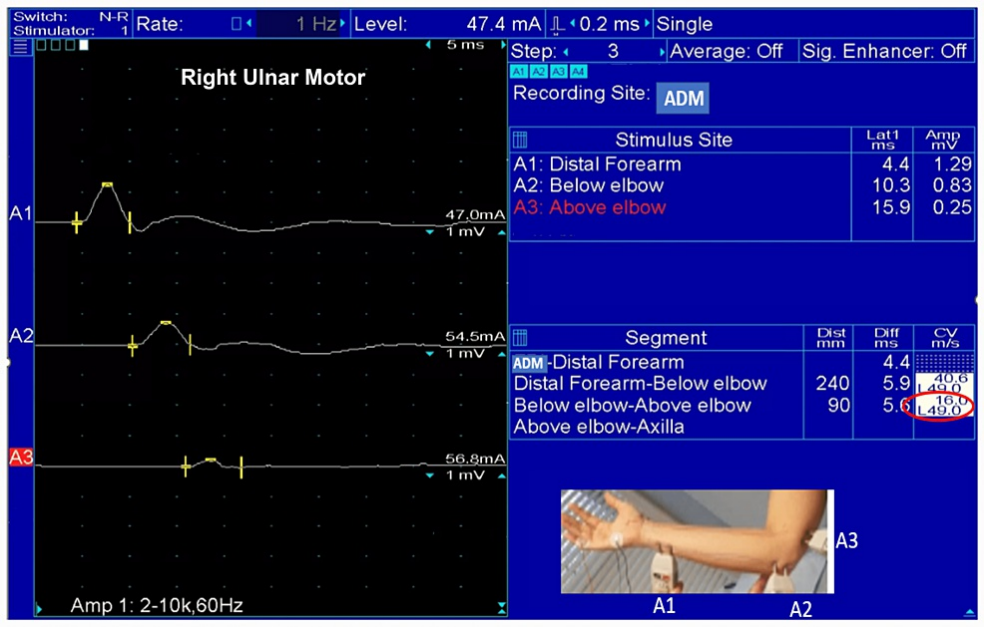
Motor Conduction Study of the Ulnar Nerve Motor conduction study of the right ulnar nerve showing marked focal slowing (16 m/s) at the elbow ADM: abductor digiti minimi

All patients had focal slowing of the motor conduction of the ulnar nerve across the elbow (Figure 1).

Sensory potentials were either absent or of a low amplitude over the digital and/or dorsal cutaneous branches of the ulnar nerve in all patients. Ten (56%) patients had evidence of denervation of the abductor digiti minimi (ADM) and first dorsal interosseous (FDI), evidenced by the presence of fibrillations and positive sharp waves on needle EMG. There were decreased motor unit recruitment in 17 (94%) patients and large-amplitude, wide-duration polyphasic motor units in 15 (83%) patients.

The EDX studies determined that the ulnar nerve neuropathy was located at the elbow in all patients. The abnormalities were suggestive of focal demyelination, conduction block, and axon loss (motor/sensory/both) in varying combinations. Focal demyelination at the elbow was documented in all patients.

Ultrasound studies

**Figure 2:**
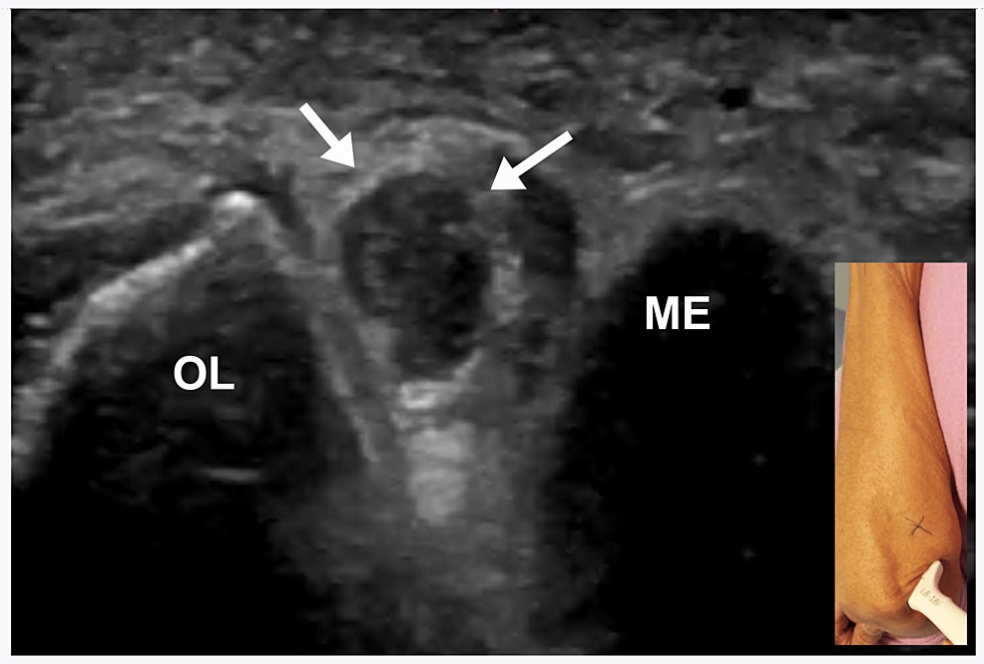
Ultrasound Study of the Ulnar Nerve at the Elbow Short-axis view of the right ulnar nerve (oblique arrows) at the elbow showing an increase in the cross-sectional area of 16 mm^2^ (normal: <10 mm^2^) ME, medial epicondyle; OL, olecranon

Fourteen (78%) patients underwent US studies. Twelve (86%) patients showed an increase in the CSA of the ulnar nerve at the level of medial epicondyle (Figure 2).

Six (43%) had a hypoechoic ulnar nerve. Two (14%) US studies were normal.

Follow-up

A total of 14 (78%) patients underwent surgical intervention for ulnar nerve neuropathy following the RTSA. These procedures consisted of four with an ulnar nerve transposition, four with a CBTR and ulnar nerve transposition, three with an ulnar nerve decompression, and one with only a CBTR. The mean duration between the RTSA and ulnar nerve surgeries was 6.8 months (range: 1½ months-20 months). Of the 14 patients who had ulnar nerve surgical procedures, only four had complete symptom resolution. The remainder continued to experience sensory abnormalities and/or weakness in the distribution of the ulnar nerve. Of the four patients who did not undergo ulnar nerve surgery, one underwent a cortisone injection to the shoulder, another was treated with physical therapy and home exercises, and two did not have any treatment. Three of these patients continued to complain of symptoms in the ulnar nerve distribution. The other one continued to have numbness and tingling of the fourth and fifth digits 14 months following the RTSA but attained complete improvement at the last follow-up 17 months postoperatively.

## Discussion

Ulnar nerve injury remote from the site of surgery should be considered in the differential diagnosis in patients with sensorimotor deficits after shoulder surgery. The first step is a thorough neurological examination to localize the site of injury. EDX studies are essential in the investigation of these patients to document the type and severity of nerve injury [2,4,13]. Nerve conduction studies can localize the site of nerve lesion and determine whether there is demyelination, conduction block, or axon loss. They are also able to differentiate distal peripheral nerve lesion from a more proximal injury at the site of shoulder surgery [15]. NCV and EMG studies can confirm the severity and distribution of axonal injuries and differentiate them from conduction block, which has a more favorable prognosis. Mild compressive neuropathies are marked by segmental demyelination, which usually presents as a conduction block, while severe processes may exhibit axonal degeneration within 3-5 days after symptom onset [13]. The EDX tests may be repeated serially to differentiate neuropraxia from axonal injury and to document the evidence of reinnervation. In Lädermann and colleagues’ study of 41 patients (42 shoulders) who underwent either an RTSA (19 shoulders) or anatomic primary shoulder arthroplasty (23 shoulders), EMG testing was performed at a mean of 3.6 weeks postoperatively in the RTSA group [3]. Subclinical EMG changes were observed in nine shoulders, primarily of the axillary nerve; eight resolved in <6 months. No patients experienced an acute lesion of the ulnar nerve postoperatively in either the RTSA group or the anatomic primary shoulder arthroplasty group. In that study, a total of 47% of patients who had an RTSA and 4% of patients with an anatomic shoulder arthroplasty had acute postoperative neurological lesions confirmed by EMG studies irrespective of clinical symptoms [3].

Intraoperative neuromonitoring during shoulder arthroplasty has been evaluated to determine the number of intraoperative nerve alerts [10,16]. In Parisien and colleagues’ study of intraoperative neuromonitoring with 36 patients (12 with TSA and 12 with RTSA), the patients who underwent an RTSA had a higher average incidence of intraoperative nerve alerts (2.17) than those who had a TSA (0.46) [10]. Rotator cuff arthropathy and preoperative decrease in active forward flexion were independent predictors of an intraoperative nerve alert. These authors acknowledge that intraoperative neuromonitoring may not have significant clinical usefulness due to the high incidence of false-positive nerve alerts and the lack of clinical correlation with postoperative neurological deficits [10]. Additionally, this monitoring also increases the cost and length of surgery. Despite these drawbacks of intraoperative nerve monitoring, it may identify evolving neurological dysfunction and prevent its occurrence [6].

While several studies have reported iatrogenic nerve injuries after TSA [3,9-13,17-19], RTSA [1-12,20,21], or arthroscopic rotator cuff repair [11,22-24], a few have described an ulnar nerve neuropathy as a sequela of shoulder surgery [8,9,11,19-21]. In Hruby and colleagues’ study of 34 patients with iatrogenic nerve injuries after an RTSA, the neurological complication rate was 2.6%, reflecting 21 patients with damage to terminal nerve branches and 13 patients with a brachial plexus injury [8]. Five patients sustained an ulnar nerve injury (isolated in three, combined with a radial nerve injury in one, and combined with lateral antebrachial cutaneous branch in one). Nerve conduction studies demonstrated a conduction block at the elbow in most of these patients. These authors suggest that these injuries are due to the position and bedding of the elbow during surgery or early postoperatively with increased compression and/or traction on the ulnar nerve [8]. O’Neill and colleagues reported a patient who underwent an arthroscopic repair of a rotator cuff muscle tear of the left shoulder and sustained an ulnar neuropathy localized to the elbow due to the postoperative immobilization of the limb in elbow flexion, as confirmed by imaging and EDX studies [13]. The surgery was performed in the beach chair position, and the patient’s left arm was manually supported by surgeons and was subsequently immobilized in elbow flexion in a padded sling postoperatively. The patient complained of a painful left elbow immediately postoperatively, necessitating the removal of the sling, and the elbow was extended. EDX studies five weeks postoperatively confirmed an ulnar nerve neuropathy at the elbow. An ulnar nerve exploration and decompression were performed two weeks later. Repeat EDX studies four months after the initial shoulder surgery revealed axonal injury of the ulnar nerve at the elbow with minimal improvement of motor function. The patient continued to experience debilitating, painful sensorimotor deficits of the left hand three years later.

Pandey and colleagues reported a single patient who sustained an ulnar nerve compression during an arthroscopic repair of shoulder instability with the use of an additional traction device [18]. The patient was placed in the lateral decubitus position. A 10%-30% incidence rate of transient neuropraxia is associated with the lateral decubitus position [18]. The majority of nerve injuries during shoulder arthroscopy occur in the beach chair or lateral position due to inappropriate patient positioning or excess traction [18]. The lateral decubitus position is more prone to traction-related neuropraxia.

While most shoulder arthroscopic surgeries are performed in the lateral decubitus or beach chair position, the modified beach chair position is another option. This latter position is performed similarly to the lateral decubitus position yet maintains a semi-upright position and allows easy conversion to an open procedure [25]. In the modified beach chair position, arthroscopic access anteriorly is made with the surgeon standing anterolaterally or superiorly with respect to the shoulder instead of an across-table anterior approach [25]. In Hoenecke and colleagues’ study of 50 patients who underwent arthroscopic shoulder surgery while in the modified beach chair position, none experienced neurological complications such as dorsal digital nerve of the thumb or traction-induced trauma to the brachial plexus, which had previously been described for the lateral decubitus position [25]. The overhead traction bar that was used for the lateral position applied 10 lb of longitudinal traction with the arm flexed and abducted 30°. These authors acknowledged that they avoided excessive traction or hyperabduction of the arm. Furthermore, the extremities were carefully padded, and the head was in a neutral position with respect to the body using a foam headrest. All patients except one in the current study were placed in a modified beach chair position during the RTSA. The EDX studies determined that the ulnar nerve neuropathy originated from the elbow in all patients. We presume that the mechanism of iatrogenic ulnar nerve neuropathy of our cases involved the modified beach chair position where the patients’ ulnar nerves of the ipsilateral arms of the RTSA were compressed at the elbow in all cases.

While the primary mechanisms of injury during RTSA include arm manipulation, hematoma formation, arm lengthening, surgical dissection, interscalene brachial plexus block, and vascular injury, most of these do not explain injuries to the distal nerves (radial, ulnar, and median nerves) [11,13]. The mechanism of ulnar nerve injury following continued elbow flexion intraoperatively may include compression at the level of the elbow as the nerve passes deep to the cubital tunnel retinaculum [4,13,26]. In addition to nerve compression in the upper extremities following an RTSA, other etiologies for a distal peripheral neuropathy include dependency on the extremity after surgery, which may result in edema in the elbow and wrist, as well as wearing a sling after shoulder surgery, the latter of which involves prolonged elbow flexion and possibly with increased cubital tunnel pressure [11]. Additionally, wearing compressive sleeves around the forearm may lead to the compression of peripheral nerves. Several suggestions have been proposed to reduce the likelihood of perioperative ulnar nerve injuries, including the following: (1) Arm abduction should be limited to ≤90° in supine position, (2) arms should be positioned to decrease pressure on the cubital tunnel, (3) a neutral forearm position is recommended when the arms are tucked at the sides, and (4) padded arm boards should be used [26,27].

The patients in our study had padding placed on all bony prominences during the RTSA. In the modified beach chair position, padding may have been placed under the elbow to support the ipsilateral arm. We speculate that the mechanism of ulnar nerve neuropathy after the RTSA in our patients may involve the protective padding, inadvertently causing pressure against the ipsilateral ulnar nerve at the elbow when the arm is in traction. In this respect, we recommend less aggressive padding of the ipsilateral elbow when this surgery is performed. We also acknowledge that the ulnar neuropathy may have been caused by a postoperative sling with the arm in a hyper-flexed position or subject to direct pressure at the cubital tunnel. 

 While ulnar nerve injuries following shoulder surgery have seldom been reported, they are exceedingly rare after an RTSA. In the few patients who have been previously described in the literature, EDX studies were infrequently performed. All patients in our series had focal slowing of the motor conduction of the ulnar nerve across the elbow. Sensory potentials were either absent or of a low amplitude over the digital and/or dorsal cutaneous branches of the ulnar nerve in all patients. The ulnar nerve neuropathy was located at the elbow in all 18 patients. Of the 14 patients who underwent US, 12 patients showed an increase in the CSA of the ulnar nerve at the elbow. While patients who sustain nerve injuries after an RTSA usually experience symptomatic relief within several weeks as described in the literature, most of these patients incur brachial plexus or axillary nerve injuries and not ulnar nerve injuries. Our study demonstrates the high number (14/18) of patients with an ulnar nerve neuropathy after an RTSA who underwent a subsequent ulnar nerve transposition and/or CBTR. Of the 14 patients who underwent ulnar nerve surgical procedures, only four attained complete symptom resolution. These findings reflect the lasting burden of ulnar nerve neuropathy following an RTSA in many of these patients.

The strength of the current study is the large number of patients who sustained ulnar nerve injury following an RTSA, confirmed by EDX studies. To our knowledge, our study is the first to highlight US findings in these patients. US has the potential to provide clues to the cause of ulnar nerve neuropathy, although rarely. We have been able to detect schwannomas and cysts (intraneural and extraneural) compressing the nerve on a few occasions in the past. Whenever possible, we perform US of nerves after the EMG study as it is available in our laboratory. However, in the present group of patients, US study did not contribute to the management. This study is likely to increase the awareness of ulnar nerve neuropathy in the settings of an RTSA and take special precautions to avoid perioperative injury to the ulnar nerve at the elbow. A limitation of the current study is its retrospective nature. Prospective studies will be important to prevent perioperative ulnar nerve injury during an RTSA.

## Conclusions

Surgeons should have a high index of suspicion for an ulnar nerve neuropathy when patients experience sensory and/or motor symptoms in the hand following an RTSA. EDX and US studies are valuable modalities in diagnosing and managing patients who have sustained iatrogenic ulnar nerve injury during an RTSA.
